# Racial and gender differences in missing children’s recovery chances

**DOI:** 10.1371/journal.pone.0207742

**Published:** 2018-12-31

**Authors:** Arnout van de Rijt, Hyang-Gi Song, Eran Shor, Rebekah Burroway

**Affiliations:** 1 Department of Sociology, Utrecht University, Utrecht, the Netherlands; 2 Department of Sociology, Stony Brook University, Stony Brook, NY, United States of America; 3 Department of Sociology, McGill University, Montreal, Canada; Mälardalen University, SWEDEN

## Abstract

We inquire whether there are race and gender differences in the recovery of missing children. We argue that race and gender differences may arise due to differential media attention, socio-economic background and police resources. Datasets used in previous research lack the representativeness and longitudinal character necessary for probing victim demographic effects on recovery success. Here we use official New York State Division of Criminal Justice Services records of all children reported missing in the period 2007–2010 containing exact dates of disappearance and recovery. In event-history analysis of these data we find that missing boys and girls have comparable daily recovery chances. Black children, however, on average remain missing longer and are more likely to still be missing by the end of our observation period than non-black children.

## Introduction

Law enforcement agencies entered a total of 464,324 missing children reports into the 2017 database of the U.S. National Crime Information Center [1, 2]. Some of these children were abducted by a family member, acquaintance or stranger, while others were lost because they wandered off, were accidentally left behind, or ran away [2]. Although most children who go missing in the U.S. are ultimately located and returned home, each year thousands remain unrecovered.

We ask if the race and gender of missing children are associated with their daily recovery chances. To the best of our knowledge, this study is the first to ask and investigate this question. There are three reasons to suspect racial and gender differences in recovery chances. First, previous studies have found race and gender differences in the likelihood and intensity of media coverage and public attention, with non-African-American girls receiving a disproportionate share [3–12]. If media coverage positively impacts recovery chances, we would expect non-African-American girls to be more quickly found, all else equal. Second, some minority children, in particular African-American children, are more likely to come from socio-economically disadvantaged families and neighborhoods [13], which may limit resources available to parents and law enforcement for recovery efforts. Third, some have found legally irrelevant factors such as victim race and gender to nonetheless impact police responses [14]. In missing children cases, such variable responses could translate into variable recovery rates.

A quantitative assessment of the effects of race and gender on recovery rates requires a representative sample of missing children cases with data on disappearance and recovery dates so that length of disappearance can be accounted for. Past studies have used national FBI statistics on numbers of missing children cases, which lack this temporal information on disappearance and recovery. Moreover, FBI statistics used in past studies lump together many kinds of missing children cases, the vast majority of which are runaways. Here we draw on a comprehensive dataset confidentially shared with the authors by the New York State Division of Criminal Justice Services containing for all missing children cases information on disappearance and recovery timing, gender and race, and case type (runaway, lost, or abduction).

## Theory

We propose three theoretical mechanisms through which missing children’s race and gender may impact their recovery chances. The purpose of this theory section is not to identify competing mechanisms for empirical testing, but rather to base the expectation of racial and gender differences in prior literature and theory.

### Mechanism 1: Disproportionate public attention to missing white girls

One mechanism through which some missing children may be more readily found is media coverage. Missing children are often featured in the news because such stories provoke emotional responses toward those who are viewed as pure and innocent victims. However, public interest in missing children and media coverage of their cases are highly unequally distributed. Certain famous incidents receive national and even international attention for months on end, while others are entirely ignored [5–8].

An extensive body of research suggests that disparities in media coverage are patterned according to social categories such as ethnicity and race [15–19], gender [20–34], religion [35–37], and sexuality [38–40]. A general finding is that in each of these categories minorities receive less coverage. However, while also women are generally underrepresented in the media, some have found women to be covered more extensively when they are victims [31, 41–43]. Scholars have theoretically accommodated this reversal in terms of chivalry and paternalistic attitudes of journalists and audiences: Women are extended more compassion and attention because of their presumed weakness and need for protection [44, 45].

Racial and gender disparities in news coverage have also been identified in various studies for the specific case of missing children. This research is informed by the “missing white girl syndrome” conjecture that media allocate disproportionate time and resources to the coverage of missing white girls, anticipating greater public interest in these cases [3, 5]. Several studies identify large coverage differences consistent with this hypothesis among small, non-representative samples of missing women [4, 6, 7, 9]. [8] carry out a large-scale test of the hypothesis using FBI data on missing children. They compare the percentages of black, non-black, male, and female missing children to the corresponding percentages of children who are featured in evening newscast reports. They find that non-black children are indeed significantly more likely to receive coverage than non-white children. Missing girls, on the other hand, are *less* likely to be in the news than missing boys. The finding of [8] for race is later replicated in [10] using alternative statistics on missing children. However, [10] find neither under- nor over-representation of girls in media coverage. [46] compare percentages of boy and girl abductions in the 1999 National Incidence Studies of Missing, Abducted, Runaway and Thrownaway Children with percentages in newspapers and do not find significant differences in the frequencies of articles about abducted girls and boys either, but do find that articles on female abductions of girls tend to be longer. [11] compares FBI statistics to coverage data from four major news websites and finds significant bias in favor of white and female missing persons, consistent with the missing white girl syndrome. Another study finds that missing children in Louisiana have more and longer articles written about them if they are white and female, providing further support for the hypothesis [12]. Overall then, the evidence clearly shows that missing white children receive more coverage, while the evidence regarding the role of gender is mixed.

Racial and gender differences in news coverage of missing children may translate into differential recovery efforts by the public and law enforcement, or in differential success of those efforts [3, 5, 8, 10, 12]. In [5] the Department of Justice is quoted stating that “Intense, early media coverage ensures that people will be looking for your child”. The notion that police recovery efforts are more effective with greater news coverage is reinforced in interviews with police officers [47].

### Mechanism 2: Socio-economic background conditions resources available for recovery

Prominent examples of missing children are characterized not just by extraordinarily extensive media coverage but also by the very wealthy families from which they were separated [48]. Resources of parents, neighborhood, and police to mobilize search efforts may improve recovery chances. Such resources will covary with the race of missing children, with African-Americans having the least. Black households in the United States in 2010 had a median income of 32,000 in contrast to a median income of 53,000 among non-black households, with 27% of black households living in poverty compared to 14% non-blacks [13].

In addition, the scope and effectiveness of police efforts may be expected to be greater in rich neighborhoods, which tend to be predominantly white. Poorer neighborhoods tend to have higher crime rates [49] and rates of police misconduct vary with poverty and racial composition of neighborhoods [50]. In addition, missing children from wealthy families may receive more extensive police attention because of parents’ greater ability to compel police to dedicate time and effort, through donations, network connections to those deciding on promotions, or other forms of power use.

Resources for recovery will be comparable for missing boys and girls, as socio-economic background does not covary with gender. Therefore, this theoretical mechanism does not suggest a gender difference in recovery rates of missing children.

### Mechanism 3: Race- and gender-dependent law enforcement responses

Conflict theories of crime [51–54] and behavior of law theory [55, 56] suggest that police responses to crimes depend on legally irrelevant features of victims such race and gender. The stratification hypothesis derived from Black’s behavior of law theory [55] is that the “amount of law” individuals receive is positively determined by their status in society. Applied to missing children, the prediction becomes that law enforcement places less effort in the recovery of girls and black children than that of boys and white children.

The empirical literature on stratification in crime finds some support that cases with black and other minority victims are less rigorously pursued. [57] reports that in sexual assault cases involving black offenders and white victims the offender received more serious charges, longer sentences, and higher rates of felony case filings, executed sentences, and state penitentiary incarceration, but did not involve higher arrest rates or percentages found guilty. [58] finds in a national survey that police respond more quickly and exert more effort in robberies and cases of aggravated assault involving white victims and black perpetrators. [59] finds that in survey data that police response times in assault, robbery and rape cases is shorter when victims are white and follow-up effort is more extensive. In two large studies of homicide cases, clearance is found more likely when the victim is white [60, 61]. [14] reports that among 13 studies of homicide arrests, 9 found racial/ethnic differences in police clearance rates consistent with the stratification hypothesis, and 4 found no differences. [62] finds no victim race differences in the chances of police clearance in archival data on cyberbullying cases. [63] and [64] find in archival data on sexual assault complaints that arrest decisions do not vary by victim race. [65] find that racial differences in clearance of sexual assault cases are attributable to black victims’ inability or unwillingness to cooperate with police. While overall these results are weakly consistent with the stratification hypothesis, they do not demonstrate police discrimination. For example, it may be that greater poverty, higher crime, and lack of collective efficacy in some neighborhoods and districts with greater minority presence (e.g. [66]) limit the amount and effectiveness of police resources that can be spent on any one case.

With regards to victim gender, findings violate the stratification hypothesis. In no study are male victims found to be preferentially treated. [58] finds that female survey respondents report quicker police response times in robbery and aggravated assault cases. [61] finds that homicide case clearance is more likely with female victims. [14] reports that of 11 studies that examine the effect of victim gender on homicide case clearance, 5 find that cases with female victims are more likely cleared while in 6 cases no difference is found. [62] does not find any victim gender difference in the likelihood that a cyberbullying case is cleared.

## Data & methods

Our research was approved by the Stony Brook University Human Subjects Committee (IRB), project #390584. Most missing children data used in prior studies are aggregate counts, such as numbers of girls and boys of different race and ethnicity recorded as missing in official statistics [8, 10, 11]. These statistics lack temporal information on the timing of disappearance and recovery of each child that is necessary for estimating daily rates of recovery. In other studies, names and demographic characteristics of missing children are extracted from online databases [12]. Online databases typically provide date of disappearance but not recovery, as cases are removed from the website when children are recovered. They thus oversample hard-to-solve cases, and a previous study found that even among those cases, not every active police record had a match [12] (see page 673). We draw from a unique dataset that contains records on all children reported missing in New York State between 2007 and 2010. The data is provided by the New York State Division of Criminal Justice Services (DCJS). The opinions, findings, and conclusions expressed in this publication are those of the authors and not those of DCJS. Neither New York State nor DCJS assumes liability for its contents or use thereof. The dataset includes information on the day a child was reported missing to the police, the day the child was found or the day the record was last updated. It also specifies the race and gender of missing children, as well as the circumstances under which they went missing (e.g., abductions, runaway cases, etc.).

The dataset contains information for 81,136 missing episodes. We exclude 37,767 possibly invalid cases from our analysis with case cancellation codes “missing person record determined to be invalid or no longer sought” or “purged due to failure to validate”. DCJS indicated to us that the first cancellation code is often selected by agencies in true recoveries of valid missing children cases. Our results are of similar size and the same degrees of statistical significance when these cases are included in the analysis. Here we report on analyses of the remaining 43,379 missing episodes, involving 31,232 unique children. Tables 1 and 2 show descriptive statistics on respectively cardinal and categorical variables used in the analysis. An important limitation these data share with other police data used in past work is that they do not differentiate Hispanic children. Whether a child is Hispanic or not is an optional entry in missing children police reports, and in practice rarely used. When used, the corresponding race category is predominantly white. In the subsequent analysis we therefore follow [8] and [11] in dichotomizing race into black and non-black children, where non-blacks include Hispanic children. Black children make up 40% of the children whose race is known. This is somewhat higher than the national figure of 36% [67], reflecting the larger percentage of blacks residing in New York State (18%) than in the United States as a whole (13%). Girls comprise 56% of all missing children cases in New York State. This overrepresentation of girls is comparable to national estimates in which 53% of missing children are girls [67]. The vast majority of children in our data are eventually recovered alive. But, time to recovery varies significantly, and 229 children had not been recovered by the end of our observation period (end of 2010).

**Table 1:** Descriptive statistics for cardinal variables.

Variable	Mean	St. Dev.	Min.	Max.	*N*
Number of episodes per child	1.6	1.3	1	26	31,232
Duration of episode (in days)	30.8	154.7	1	2,746	43,379
Year of disappearance	‵08.4	1.9	‵07	‵10	43,379
Age at disappearance (in years)	15.0	1.9	0	17	43,379

**Table 2:** Descriptive statistics for categorical variables.

Variable	*N*	% cases
**Race / ethnicity**	**43,379**	**100.0%**
Black	16,990	39.2%
White (including Hispanic)	24,410	56.3%
Asian	425	1.0%
Native American	167	0.4%
Unknown	1,387	3.2%
**Gender**	**43,379**	**100.0%**
Female	24,379	56.2%
Male	19,000	43.8%
**Circumstances**	**43,379**	**100.0%**
Abduction	297	0.7%
Lost/wandered away	1,015	2.3%
Runaway	38,378	88.5%
Unknown	3,689	8.5%
**Outcome**	**43,379**	**100.0%**
Never found	229	0.5%
Found	43,150	99.5%

We measure duration as the number of days between the day the child was reported missing and the day s/he was recovered. If a child is never found, we measure duration as the number of days between the day the child was reported missing and the last observed day in the dataset. Episodes on average last 31 days, but duration varies greatly, with the standard deviation being 5 times the mean. Cases in which the child was found include police recoveries, cases where the child deceased, and instances in which the child voluntarily returned home.

The New York State data differentiate between different types of missing children cases. Past studies of missing children media coverage often do not differentiate between case types [8, 10, 11]. Yet aggregate statistics are dominated by children who ran away from home, while media coverage will naturally overrepresent more spectacular cases, such as abductions. If race and gender correlate with case type, then it is essential to control for case type in assessing claims about race and gender effects on media coverage and daily recovery chances. As in national statistics, also in the New York State data most cases are runaways. Children who get lost or wander away are less common. Abductions are exceedingly rare.

## Results

**Fig 1:**
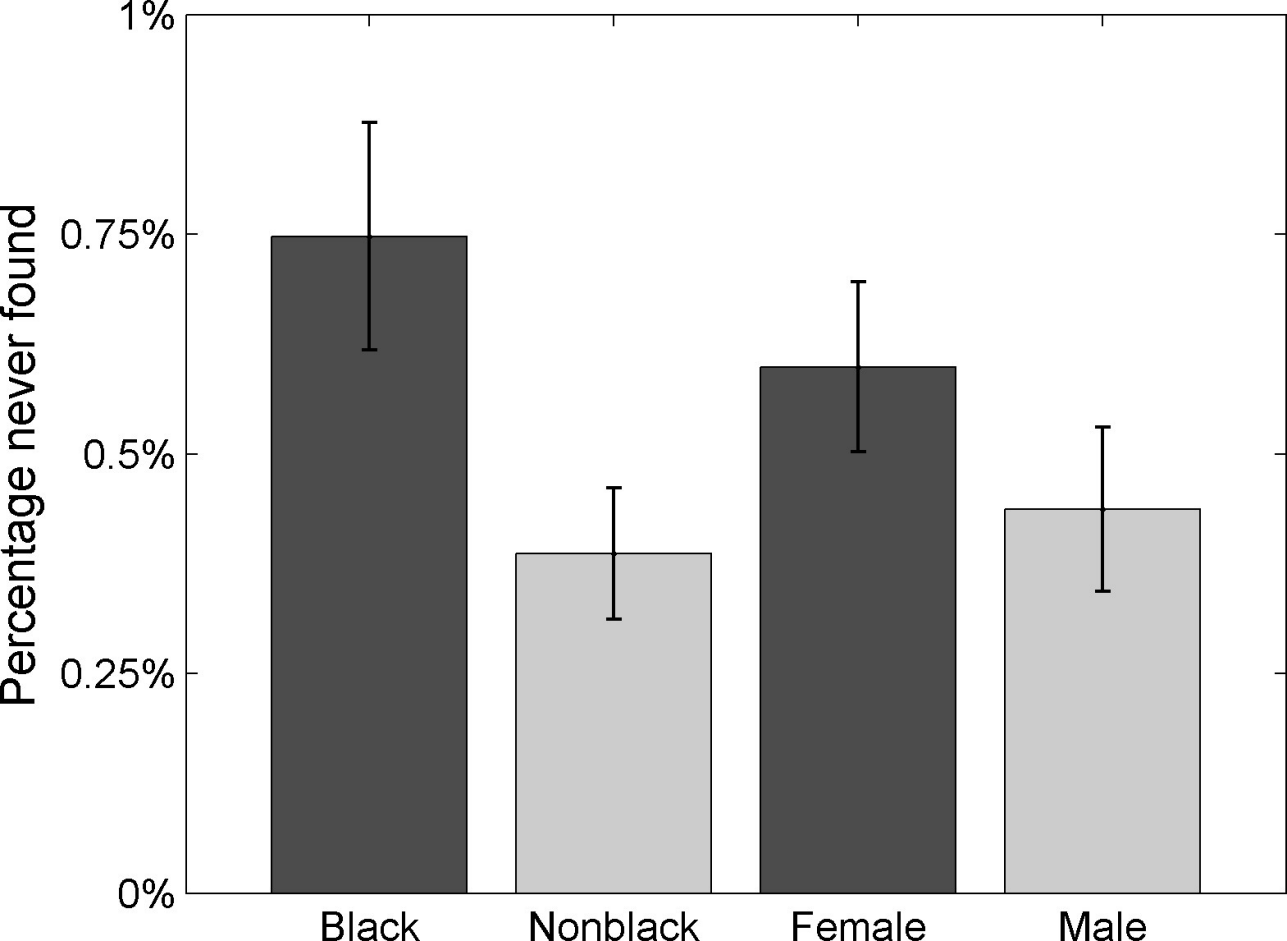
Percentage of children never found, by race and gender.

Fig 1 shows that black children are about twice as likely as other children to remain missing by the end of our observation period. Regarding the gender of missing children, we find that missing girls are slightly more likely to remain missing by the end of our observation period than missing boys. A G test finds both the racial and the gender difference statistically significant at the 95% level.

**Fig 2:**
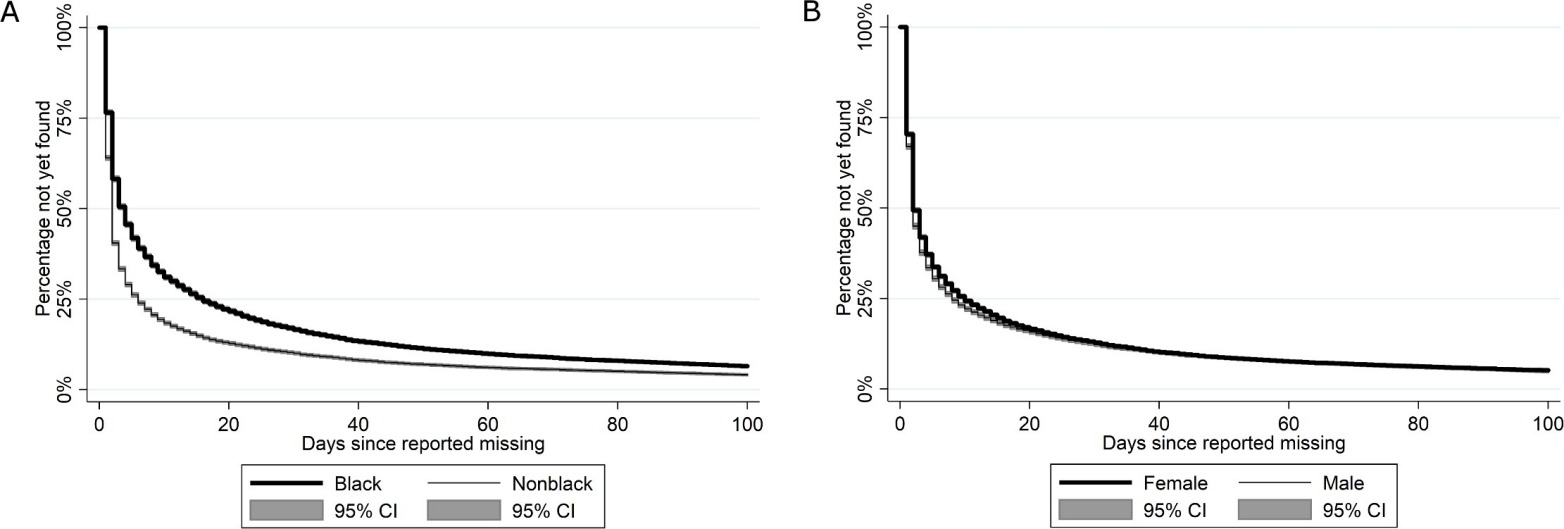
Percentage not yet found by race, gender, and number of days missing.

Next, we explore whether these race and gender differences can be explained by variation in case-specific factors. First, there may be ethnic / racial and gender differences in the length of time for which children are missing. The analysis in Fig 1 does not differentiate among the 99% of cases in which children were ultimately found in terms of how quick the recovery was. To account for potential racial and gender differences in the duration that children are missing and the possibility that children who are later found are censored as they fall outside our observation period, we use event history analysis techniques [68]. Each episode in the event-history analysis starts on the day a child goes missing and ends when the case is resolved. At the start of the episode the clock is reset to 0, so that we analyze recovery chances as a function of the number of days since the most recent date on which a child went missing. The event variable ‵recovered’ is coded 1 if the episode ends with a recovery and coded 0 if the episode is right-censored because the case was still open at the time the data was collected. For the 43,379 episodes, representing 31,232 unique children, Fig 2A and 2B show non-parametric Kaplan-Meier estimates of the recovery rates of black versus non-black children respectively girls versus boys [69]. Fig 2 shows that the probability that a child is still missing by a given day is consistently and significantly higher for blacks than for non-blacks at the 95% confidence level. Fig 2 also reveals that boys are recovered slightly more quickly than girls, and that this difference is statistically significant.

There are several variables that may confound these race and gender effects for which our data provide measures. First, there may be racial / ethnic differences in the age in which children go missing. Older kids are more often missing because they leave deliberately, are more able to return if desired, and their cases may have less priority in the eyes of police or the public, all of which may impact the likelihood of recovery. The circumstances under which children go missing may also vary by race and gender. Runaways may tend to happen in poor neighborhoods and as a result more often concern black children. To account for such case-specific differences we regress the daily ‵hazard’ or likelihood of recovery in a series of Cox proportional hazard models [70] on the gender and race of the missing child as well as control variables.

Table 3 reports results from three Cox models (Models 1, 2, and 3). These models control for the age at which a child goes missing, the number of times a child has gone missing before, and binary variables for different circumstances under which a child goes missing. The table displays logged hazard rates and cluster-adjusted standard errors in parentheses. A positive coefficient means that the predictor variable increases the hazard rate of recovery. Models 1 and 2 reported in Table 3 include all episodes. Model 1 includes only the main effects of gender and race which have our theoretical focus. Model 2 additionally includes control variables, allowing us to evaluate whether the main effects of gender and race are attributable to otherwise uncontrolled situational features. In Model 3, we analyze only the last episode of a child, excluding earlier episodes involving the same child. The purpose of this model is to verify that our results do not depend on the disproportionate role in our estimates of a small number of children who regularly go missing. Model 4 contains the same predictors as Model 3 but is a lognormal survival model instead of a Cox proportional hazard model. The coefficients are negative logged time ratios instead of logged hazard ratios. A positive coefficient means that the predictor variable decreases the expected time until recovery. The purpose of Model 4 is to evaluate whether the effects of race and gender depend on the assumption made about the shape of the survival function. The lognormal survival model is shown because it fit best among a range of common alternatives, including Weibull, Gompertz, loglogistic and exponential.

**Table 3:** Survival models regressing recovery hazard of missing children on race, gender, age, episode number, and circumstances.

	Model 1: Cox; all cases	Model 2: Cox; all cases	Model 3: Cox; only last case	Model 4: lognormal; only last case
	ln(hazard ratio) [z]	ln(hazard ratio) [z]	ln(hazard ratio) [z]	-ln(time ratio) [z]
Black	-.27 [-28.9] ***	-.28 [-29.2] ***	-.30 [-28.3] ***	-.65 [-33.2] ***
Female	-.06 [-6.5] ***	-.06 [-6.2] ***	-.06 [-5.6] ***	-.12 [-6.2] ***
Age		-.04 [-10.6] ***	-.04 [-9.0] ***	-.09 [-14.6] ***
Episode number		.00 [0.1]	n.a.	n.a.
Circumstances (baseline: Abduction)				
Lost/wandered away		1.21 [12.1] ***	1.16 [11.1] ***	2.20 [-11.5] ***
Runaway		1.14 [12.4] ***	1.07 [11.3] ***	1.98 [-10.9] ***
Unknown		1.15 [12.3] ***	1.11 [11.7] ***	2.08 [-11.3] ***
Number of unique children	31,232	31,232	31,232	31,232
Number of episodes	43,379	43,379	31,232	31,232
Number of recoveries	43,150	43,150	31,004	31,004
Wald *χ*^2^ (df)	871 (2) ***	1001 (7) ***	913 (6) ***	1369 (6) ***

Notes: Cluster-adjusted z-scores in parentheses.

*** = *p* < .001.

** = *p* < .010.

* = *p* < .050.

In each of the models in Table 3 the daily chances that a child is found given that is has so far not been found are estimated to be lower for black children than for non-black children. Also, the hazard rate for girls is lower than for boys. The coefficient of -.27 in Model 1 suggests that the hazard rate at which black children are found is 24% (1-exp(-.27)) lower than the rate at which non-black children are found. Model 1 also shows that girls are found at a rate that is 6% lower than that of boys. Model 2 shows that these gender and race effects remain significant and of approximately the same magnitude when control variables are included. Model 3 shows that the inclusion of only the most recent episode per child does not impact estimates. Model 4 shows that the results are robust to alternative assumptions about the baseline survival function.

The effect of age is significantly negative in Models 2–4, indicating that older children are less readily found. In analyses not shown here we added dummy variables for different age groups, where we found that very young children, of whom there are few cases, form an exception, as they are the least likely to be recovered on a given day. These analyses also show that the gender and race effects do not depend on whether age is estimated in such piecewise fashion or as a single linear term. The inclusion of age as a predictor does not alter the magnitude or direction of either the race or gender effect, suggesting that race and gender differences in daily recovery chances are not due to differences in the typical age at which children go missing. The null effect of episode number in Model 2 suggests that how often a child has gone missing before does not impact its likelihood of recovery. The effects of the circumstances under which a child went missing show that abduction cases are much less likely to be resolved by a given time than other cases. Runaway cases, cases in which a child was lost or wandered away, and unknown cases all have comparable daily rates of recovery.

Table 3 leaves open the possibility that the race and gender effects on recovery rates are specific to certain case types. For example, media, public and police attention may be greater in abduction cases, which may generate different gender and race effects. Also, runaway cases are more often concluded with a child returning home voluntarily, an outcome that family and law enforcement may have less control over. To explore these possibilities, we estimated a model with control variables for each case type separately. The four sets of model estimates are shown in Table 4. Table 4 reveals that the effect of race is of similar magnitude in all models and statistically significant in the prediction of daily recovery in all missing child cases except abductions. The effect of gender is of similar size in lost, runaway, and unknown cases and only significant in the prediction of runaway cases. The significance levels of the effects of race and gender clearly covary with the frequency of cases in each case type category. To test for significant variation in the magnitude of the effects of race and gender across case types we estimated a joint model with interaction effects between race and case type and then calculated Wald, BIC and AIC statistics for goodness-of-fit comparison. None of the effects of individual interaction terms was found statistically significant. All three goodness-of-fit tests favor models without gender interaction effects. The AIC and Wald tests favor models with race interaction effects, while the BIC test does not.

**Table 4:** Survival models regressing recovery hazard of children who were abducted, were lost, ran away or whose status is unknown.

	Model 5: abduction; all cases	Model 6: lost/wandered away; all cases	Model 7: runaway; all cases	Model 8: unknown; all cases
	ln(hazard ratio) [z]	ln(hazard ratio) [z]	ln(hazard ratio) [z]	ln(hazard ratio) [z]
Black	-.18 [-1.6]	-.27 [-4.4] ***	-.30 [-29.6] ***	-.20 [-6.4] ***
Female	.13 [1.1]	-.08 [-1.5]	-.06 [-6.4] ***	-.05 [-1.6]
Age	.04 [4.3] ***	-.03 [-1.7]	-.08 [-21.2] ***	.00 [.2]
Episode number	.13 [5.8] ***	-.04 [- 2.1] *	.00 [.9]	-.04 [-2.8] **
Number of unique children	290	976	27,643	3,130
Number of episodes	297	1,015	38,378	3,689
Number of recoveries	258	1,004	38,207	3,681
Wald *χ*^2^ (df)	68 (4) ***	29 (4) ***	1,240 (4) ***	51 (4) ***

Notes: Cluster-adjusted z-scores in parentheses.

*** = *p* < .001.

** = *p* < .010.

* = *p* < .050.

The age at which a child goes missing has markedly different effects on daily recovery chances depending on the circumstances under which a child goes missing. The positive effect of age on recovery chances of abduction cases is inconsistent with paternalistic patterns of response, whereby younger children receive greater attention and care, but consistent with the notion that older children are more capable of helping themselves out of a difficult situation.

The negative effect of age on daily recovery chances in runaway cases is consistent with the notion that older children are more able to effectively stay away from their parents and may also indicate that there is less effort by hosts, parents, and law enforcement to bring older children back home.

## Discussion

This article has presented the first quantitative assessment of race and gender differences in missing children’s recovery chances. Our analysis of historical New York State missing person cases showed that blacks on average remain missing longer and are more likely to still be missing by the end of our observation period than non-black children. The race effect was found for all missing children cases, except abductions. However, the effect of race on recovery in abduction cases was found to be no weaker than the race effect in other cases, and it is possible that the small number of abductions in the data prevented an effect of similar magnitude from showing up.

The analysis also showed an effect of gender on recovery rates, but this was smaller, at 6% lower rates for girls compared to boys. The effect was only significant for runaway cases, but the magnitude of the effect did not significantly vary by case type. We conclude that the recovery chances of boys and girls are similar, with a slight overall difference in favor of boys.

Our analysis could not identify the mechanisms driving these effects. Nonetheless, we can draw some tentative conclusions regarding the theoretical mechanisms on the basis of the racial and gender effects found. In our theoretical analysis we proposed three mechanisms through which race and gender of missing children may impact their likelihood of recovery: The greater news coverage that white girls receive may aid search efforts, the resources available for search efforts involving black children may be more limited, and law enforcement may exert greater effort in cases involving white boys. These mechanisms unequivocally imply lower daily chances for black children to be found while being ambiguous about the net effect of gender. This theoretical implication is supported by the empirical analysis. The three theoretical mechanisms together do not yield a clear hypothesis regarding the effect of gender on daily recovery chances. Missing girls are on the one hand expected to receive more media exposure than missing boys, but on the other hand the stratification hypothesis predicts they receive less police effort (even if data on other types of police cases do not support this). The empirical results indicate similar recovery chances of girls and boys.

### Limitations and future directions

One important limitation of the present study is that the police records it draws on do not systematically indicate the Hispanic ethnicity of missing children. As a result, White and Hispanic children were mixed into a single race category, even though many of the mechanisms predicting an African-American disadvantage also predict a Hispanic disadvantage. Under this assumption, the race difference found in the present analysis would be interpreted as an underestimate, as it would then be stronger if Hispanics were effectively filtered out of the White category.

Another key limitation is our lack of measures of mechanisms through which race and gender differences in the recovery chances of missing children could come about. While the findings of a considerable race effect and a minimal gender effect are broadly consistent with our theoretical analysis, the empirical support remains highly tentative and does not allow unambiguous interpretation. A promising direction of inquiry is identifying the mediating effect of media coverage in the relationship between race and gender and likelihood of recovery, which may help isolate the public attention mechanism. Past studies have done ecological analyses in which gender- and race-specific rates of media coverage were related to corresponding rates of children going missing [8, 10–12, 46]. One could extend such analyses by comparing these national or state-level data with rates of missing child recovery. However, such analysis would have limited causal inference potential because one would lack important controls such as the type of missing child case (runaway / lost / abducted) and one would not know if the children mentioned in the media are representative samples of underlying subpopulations. An alternative individual-level approach is to identify news coverage of specific child names taken from official records of missing children and then compare recovery rates. [12] queried the names of 55 missing children in Louisiana State in news databases and search engines, but this did not provide sufficient power for an analysis of recovery. We attempted to scale this method using large-scale newspaper databases but found that in fact media cover very few cases and that these instances are hard to link to identity databases. In these analyses we tried various basic search strategies in which queries combined first and last names from our records with terms like “missing” and “abducted”. Our attempts generated zero matches for most missing children names, and virtually all matches found were false positives. Assessing the extent to which race and gender differences in missing children’s recovery chances can be attributed to differential media attention is thus a methodological challenge that we leave for future research.

Future studies may also seek to provide more direct evidence for a resource mechanism driving differences in recovery rates. A limitation of the data used here is that they lack a measure of the socio-economic background of the children, or a precise enough measure of household location that would allow an assessment of the role of socio-economic status. Scholars may seek to match police records with data from other sources containing such additional measures. Such measures would allow one to more directly test to what extent race effects are produced by socio-economic differences, e.g. through limits on available resources for recovery.

Finally, while the results presented here are consistent with conflict theories of crime and behavior of law theory, we have no evidence that law enforcement efforts actually vary by race or gender, as these theories would suggest. One direction for future research may be for researchers to attempt to measure the overall share of the budget expended by police on various missing child cases. Departments, by providing detailed case records or officer testimonies that would allow such an evaluation, could help verify that they work equally hard for the safe return home of every missing child.
